# The health-performance framework of presenteeism: A proof-of-concept study

**DOI:** 10.3389/fpsyg.2022.1029434

**Published:** 2022-11-15

**Authors:** Caroline Biron, Maria Karanika-Murray, Hans Ivers

**Affiliations:** ^1^Department of Management, Faculty of Business Administration, Université Laval, Québec, QC, Canada; ^2^VITAM Research Center on Sustainable Health, Québec, QC, Canada; ^3^Lancaster University Management School, Lancaster University, Lancaster, United Kingdom; ^4^Department of Psychology, Nottingham Trent University, Nottingham, United Kingdom; ^5^School of Psychology, Université Laval, Québec, QC, Canada

**Keywords:** presenteeism, health-performance framework, health, productivity, job stressors

## Abstract

There is emerging research that considers presenteeism as a neutral behavior that has both positive and negative predictors and outcomes for individuals and organizations. This neutral perspective diverges from the traditional negative view of presenteeism and is aligned with the Health-Performance Framework of Presenteeism (HFPF) in which presenteeism is considered to be an adaptive behavior that aims to balance health limitations and performance demands. This proof-of-concept study aims to investigate the existence of different profiles of presentees based on their common health problems (mental and physical) and performance, and differences in attendance and job stressors among these subgroups. Latent profile analysis with 159 clerical employees and managers from the UK private sector supported the HPFP and revealed four profiles: those reporting a good health and high performance were labeled *functional presentees* (who represented 19% of the sample), those with poor health and low performance were the *dysfunctional presentees* (14%), those with relatively high performance but poor health were labeled *overachieving presentees* (22%), and those with average scores on both dimensions were the *average Joe/Jane presentees* (45%; a new profile based on this sample). There was no profile in the present sample that corresponded to *therapeutic presenteeism*, characterized by low performance but relatively good health. Although *average Joe/Jane presentees* were comparable to *functional presentees* in exposure to most job stressors, they reported poorer pay and benefits, and more health problems than the latter. *Average Joe/Jane presentees* reported the lowest number of days of presenteeism. No difference was found in absenteeism across profiles, highlighting difficulties in measuring presenteeism using a count-measure, since three profiles presented a similar number of days of presenteeism yet contrasted health-performance configurations. *Dysfunctional presentees* were systematically more exposed to job stressors compared to *functional presentees*. The results support the HPFP proposition for different subgroups of presentees who are influenced by their work environment. The study takes a person-centered approach, disentangle presenteeism from the total count of presenteeism days, offering implications for management and intervention practice. Presenteeism can have a bright side and be functional in certain contexts when the appropriate resources are available.

## Introduction

Presenteeism is defined as the behavior of working while ill (Ruhle et al., 2019). This behavior is adaptive and “serves the purpose of balancing health constraints and performance demands, especially in the case of non-contagious health problems” (Karanika-Murray and Biron, 2020, p. 244). It is a global phenomenon documented in many countries with prevalence reported to range from 30 to over 90% in different studies (Karanika-Murray and Cooper, 2018; Lohaus and Röser, 2019). In the UK, Kinman (2019) reports that 50–70% of workers attend work while ill at least 1 day per year. Because of these rates, research interest in this topic is increasing fast. For example, a Google Scholar search with the word “presenteeism” yielded 4,460 hits between 1996 (when the term was first coined by Cary Cooper) and 2010. The same search yielded 19,500 hits between 2010 and 2022. Despite its prevalence and the high costs for individuals and organizations, to date our theorizing is disproportionately weak, rendering our understanding of presentees’ experiences and how presenteeism should be managed weak.

Findings from longitudinal studies concur with those of cross-sectional research on the negative effects of showing up at work while ill on individuals’ mental health (Demerouti et al., 2009), physical health (Kivimäki et al., 2005; Bergström et al., 2009; Skagen and Collins, 2016), and productivity (Zhou et al., 2016). There are two issues with this line of research. First, there are inconsistencies in these findings. For example, Collins et al. (2018) found no effect of presenteeism on well-being and performance over time, which may suggest that not all presentees experience presenteeism in the same way. The popular variable-based perspective, which looks at the antecedents of presenteeism and related outcomes, implies that all presentees experience or enact the behavior in the same way or indeed that they form a homogeneous group. Furthermore, there has been an emphasis on the negative aspects of presenteeism, or what Cooper et al. (2015) called the bad presenteeism phenomenon, thus overlooking its potential positive side. Calls for a more neutral and functional definition of presenteeism (Ruhle et al., 2019; Karanika-Murray and Biron, 2020) have led to more insightful research investigating positive motives (Knani et al., 2021; Lohaus et al., 2021) and potential benefits (Wang et al., 2022) of presenteeism. For example, evidence of positive effects for working while ill comes from Lohaus et al. (2022, 2021) who identified several categories of factors, including social norms (e.g., being liked, maintain career prospects, being loyal), financial considerations, showing endurance, and getting work done. Similarly, in a qualitative study with small enterprises, Knani et al. (2021) revealed several motives explaining why workers and managers came to work despite illness. Positive motives related mainly to personal values, avoiding isolation while being ill, feelings of accomplishment and commitment, a convivial work environment, and the possibility for work adjustments. The person-centered and positive approach is aligned with the Health-Performance Framework of Presenteeism (HPFP) developed by Karanika-Murray and Biron (2020), which has yet to be empirically tested.

The present study aims to identify profiles of presenteeism and examine differences among them. It is a proof-of-concept study that is focused on the presenteeism typology proposed in the HFPF but also expands on that to examine profile differences in attendance behavior and job stressors associated with each. As such, we hope that taking a functional approach and focusing on understanding groups among presentees will address some of the debates in the field, specifically relating to the assumptions that presenteeism is a negative phenomenon and that it is experienced in the same way by all presentees. Understanding profiles and group differences in presenteeism can support better management and targeted interventions to promote employee health and performance at work.

### Key debate: Is presenteeism inherently negative?

A key debate in the field relates to the overwhelmingly negative view of presenteeism. Ruhle et al. (2019) suggested that presenteeism should be viewed as a neutral behavior and that positive or negative antecedents or consequences should not be ascribed to it. They report on debates about the definition of presenteeism, which has tended to oscillate between two main schools of thought. First, mainly in European and Scandinavian studies, presenteeism has been defined either as the “act” of *showing* up at work with a health impairment (e.g., Aronsson and Gustafsson, 2005; Taloyan et al., 2012; Marklund et al., 2015). The COVID-19 pandemic has brought several workers into telework and recent studies show that working at home despite illness [recently labeled as “workahomism” by Brosi and Gerpott (2022)] is as prevalent and perhaps even more than when workers work physically on site (Steidelmüller et al., 2020; Biron et al., 2021). This shift calls for a definition that does not necessitate physical presence at work. Second, mainly in North American studies, presenteeism is often referred to in terms of productivity losses associated with various health impairments (e.g., Stewart et al., 2003; Goetzel et al., 2004). In this line of research, presenteeism is not measured directly but is instead inferred from participants indicating how much a health impairment has affected certain aspect of their performance or productivity at work (Johns, 2011). This view, and its related measures, can be problematic as it conflates the behavior of presenteeism with its consequences and has negative connotations. However, Ruhle et al. (2019) point out that: “Research on presenteeism should refrain from evaluating and labeling the behavior as positive or negative. Further, the definition should not imply any motives or consequences (such as productivity loss or future health impairments)” (p. 3). They therefore suggest that the definition of presenteeism as the *act* of working in a state of ill-health is more accurate. This is measured by asking presentees to indicate how many times or how many days they worked while ill over a period, usually between 3 and 12 months. Yet, although straightforward and therefore popular, count measures do not allow to differentiate among possible subgroups of presentees who have different health and performance configurations and experience presenteeism differently.

### Key debate: Are presentees a homogeneous group?

A related key debate in the field is about how presentees themselves are described and therefore how well their experiences are understood. Performance and productivity losses are often considered to be outcomes of the decision to work while ill (“I am working even though I should not and therefore not being very productive”), whereas health problems, individual’s values, pressures in the work environment, and organizational factors tend to be viewed as antecedents (“I am ill and yet I choose to work because of such and such motives”). This is in line with Lohaus and Habermann (2019) framework that highlights several person-specific (e.g., attitudes, values, health situation), work or job-related (e.g., ease of replacement, supervisor support, job demands/workload, adjustment latitude), and organizational-level variables (e.g., reward system, paid sick leave, job security). These variables can in turn be shaped by the broader context (e.g., economy, culture). They influence the individual’s decision to be absent or present, leading to several individual (e.g., health deterioration, productivity loss, exhaustion) and work/organizational consequences (e.g., higher accident rates, productivity loss). Variable-based models are comprehensive and is useful for disentangling antecedents from outcomes (variance models, which focus on explaining the maximum variance in the target variable) or understanding the chain of effects to and from a target variable (process models, which focus on what variable affects what other variable and in what order). However, variable-based models do not consider the possibility of subgroups of workers who may be affected in different ways and who may have different experiences. A suitable alternative is the person-centered approach that allows to identify subgroups of individuals who represent different configurations of several variables, including antecedents and outcomes. Person-centered research can allow us to investigate what variables predict belonging to a certain subgroup and therefore bring more clarity on the types of interventions and resources that should be deployed to foster more functional presenteeism.

The current study is offered a as way to help disentangle some of the debates in presenteeism research. Next, we summarise the proposal Karanika-Murray and Biron (2020) proposal to consider presenteeism as a function of health and performance, before developing the argument for three propositions that will be tested empirically.

### Presenteeism as a function of health and performance

**FIGURE 1:**
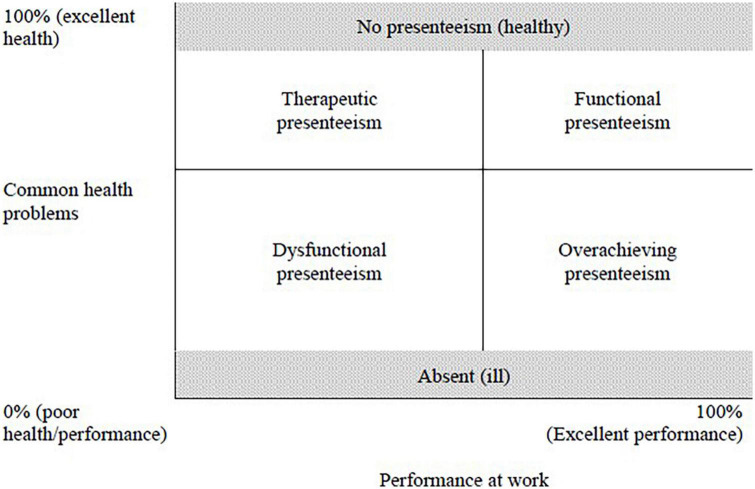
Typology of presenteeism as a function of health and performance. Reproduced from Karanika-Murray and Biron (2020), with permission from SAGE Publications.

In line with a more person-centered and functional approach to presenteeism, Karanika-Murray and Biron (2020) proposed the following definition: “presenteeism as goal-directed and purposeful attendance behavior aimed at facilitating adaptation to work in the face of compromised health” (p. 245). Their Health-Performance Framework of Presenteeism aims to unite the two schools of thought (namely, the health focus and the performance focus) and has three elements: a definition of presenteeism as an adaptive behavior, an understanding of that behavior in terms of health and performance where functional presenteeism represents a balance between the two for the individual presentee, and an ensuing 2 × 2 taxonomy of presentees that describes their health and performance experiences (see Figure 1). Here, “health” refers to common health problems (e.g., musculoskeletal disorders, stress, depression, and anxiety). We start with common health problems to understand the principles, as more severe health issues may have different adjustment demands.

Using insights from a range of related fields, Karanika-Murray and Biron (2020) emphasized that presenteeism has an adaptive function for workers who act with agency in the decision-making process (Whysall et al., 2023). Indeed, recently Lohaus et al. (2022) provided in-depth evidence that presentees make their decisions with intent and a consideration of trade-offs in the decision to work when experiencing illness. The potential for adaptation means that in order for presentees to be able to respond to both their performance requirements and the health impairments that they face, they manage their health and work resources, perhaps by either striving to protect their resources (e.g., their health, relationships at work, career development opportunities, consideration from their superior, etc.) or by obtaining new resources (see Conservation of Resources Theory, Hobfoll, 1989). Because of the variability in health conditions or impairments and performance requirements or tasks (as well as the potential combinations of these), the process of adaptation could therefore serve different purposes for different individuals. As a result, the combination of high and low health and performance requirements raises four presenteeism profiles: Functional (high performance, good health), Dysfunctional (low performance, poor health), Overachieving (high performance, poor health), and Therapeutic (low performance, good health). By adopting a functional approach and aiming to understand how health impairments and performance requirements together define the presentee’s experience, the HPFP differentiates among subgroups of presentees with different health and performance configurations.

Given the potential variability in health and performance status of workers who engage in presenteeism, it is important, for both theoretical and practical reasons, to understand the experiences of different groups of presentees. This study expands on the HPFP typology to examine the differences in attendance behavior among the four presentee profiles and the job characteristics associated with each. This knowledge supports managers and practitioners in developing more targeted and effective interventions to support both employee health and work performance.

### Performance is not uniformly affected during presenteeism and not all health conditions are equally debilitating

The argument for differential presentee profiles is supported by research that shows variability in health conditions and their impact as well as variability in performance outcomes. This makes us question whether the experience will be same for individuals with different health conditions. First, performance and productivity loss can be a potential consequence of attending work while ill, but not universally. For example, Miraglia and Johns (2016) meta-analysis showed that presenteeism is positively related to productivity losses, but not with global performance. Note that although the two terms are sometimes used interchangeably, productivity loss generally has a within-person referent, whereas performance refers to between-person differences and is used to refer to compare to other workers doing the same type of work (Miraglia and Johns, 2016). Second, different types of health ailments have been shown to have different effects on productivity (Burton et al., 1999; Goetzel et al., 2003; Lerner et al., 2004; Schultz, 2007). Health impairments can include acute (e.g., the flu), chronic (e.g., musculoskeletal problems), and episodic (e.g., allergies, migraine) physical or mental problems, as well as behaviors that are damaging to health (e.g., smoking) (Burton et al., 2004). Each type of impairment will incur a different loss of resource on the individual and their capacity to carry out their work. Similarly, variations have been reported in how different health issues are linked to performance. Whysall et al. (2018) used a cross-sectional design with 316 workers in a utility company, found that the most frequent health problems associated with presenteeism were not the same as the ones perceived to impact performance. For example, although colds and the flu were reported by the largest proportion of employees (84%), presenteeism on these days affected performance on a limited number of days (4.3 over a year), whereas hand and wrist pain only affected a small proportion of workers (6%) but impacted performance on a substantial number of days (81.6 over a year). Common health problems (stress, anxiety, and depression) were reported as the third cause for presenteeism (by 21% of their sample) and affected performance on a moderate number of days (30 over a year).

Several individual, job, and work-related factors may help to explain these differences, as studies on productivity loss during illness have highlighted. For example, Johns (2011) found that productivity loss during illness was lower for those with higher job security, for conscientious workers, and for those who could more easily be replaced at work when ill, whereas those with more pronounced neuroticism and higher family-to-work conflict reported greater productivity loss. These effects may have a temporal dimension. Specifically, Lu et al. (2013) failed to detect a long-term impact of presenteeism on performance at 2 months, although the data supported a link between presenteeism and health (physical and mental), exhaustion, and job satisfaction. Lu et al. (2013) suggested that resources (personal and social; work and non-work) act as moderators of the association between presenteeism and performance. Similarly, Wang et al. (2022) found that presenteeism had positive effect on performance evaluation 8 months later, but only when workload was high.

Overall, this evidence suggests that the variations in health conditions and performance requirements render presenteeism experiences different for different individuals. Thus, there are different configurations of health and performance that create different presentee profiles, as the HPFP suggests. A first step to understanding these profiles would be a proof-of-concept study to map them. Our first proposition is as follows.

*Proposition 1*: There are different subgroups (or profiles) among sickness presentees based on their perceived health status and performance level.

### Different presentee profiles will show different attendance patterns

As the health-performance balance will differ for each presentee, these may also affect their attendance patters and choices between attending work or taking sickness absence. Thus, variations in attendance patterns can also be expected. Several studies have shown that the severity and nature of the health impairment has an impact on both the frequency and duration of attendance behavior. For example, in their meta-analysis, Miraglia and Johns (2016) used estimated population correlations to show that presenteeism and general health status were negative correlated but also that presenteeism and depression were positively correlated. This suggests that mental health problems are perhaps not considered as a legitimate cause of absenteeism among some workers. Also, workers with a depression might be unaware or in denial of their situation, and denial in cases of depression is a well-documented area (Ketterer et al., 1996). As previously suggested by Gosselin et al. (2013), the severity, chronicity, and the type of health ailments are likely to be more or less debilitating for different individuals, and therefore likely to impact on the decision to be either absent or present. Ruhle et al. (2019) suggest viewing health as a non-dichotomous state with an individual perceiving no symptoms of illness, on the one side of the continuum, and severe health impairment or multiple ones concurrently, on the other.

The current research does not allow us to conclude whether presenteeism or absenteeism will be a choice or what patterns of attendance each type of presenteeism will be associated with. A systematic review by Skagen and Collins (2016) suggests that working through illness is associated with poorer self-reported health and increased absenteeism in the future, potentially through depletion of resources. In their study with nurses, Dew et al. (2005) found that they described their work team as “family” and their workplace as a “sanctuary,” which led them to engage in presenteeism behavior. This aligns with the qualitative research by Knani et al. (2021) in small enterprises who describes the concomitant presence of positive and negative (pressure-inducing) factors explaining presenteeism, but also some of their consequences. However, the relevant scarcity of research on the potential positive side and benefits presenteeism does now allow us to conclude whether it leads to negative outputs for all workers in the short vs. the long term, nor the reciprocal relationships between their health impairment, their performance, and the availability and usefulness of individual/work/organizational resources—and, importantly, how these lead to different attendance patterns. Yet, we can confidently expect that the combination of health limitation and performance demands will lead to different attendance choices, which is important to ascertain as the first step. This leads to our second proposition as follows.

*Proposition 2*: Different subgroups of presentees, identified based on their common health problems and level of performance, show different attendance patterns at work.

A better look and decision at how presenteeism is and should be measured is important, in view of the fact that current research does not provide a clear picture of its impact. We suggest that count measures of presenteeism (i.e., number of days) can be used to select participants who declare presenteeism, before we then look more closely at variations of health and performance and/or other factors that influence presenteeism. As previously mentioned, most research on presenteeism to-date has adopted a variable-based approach, that focuses on identifying the variables associated with presenteeism as antecedents, moderators, mediators, or outcomes of presenteeism. This variable-based approach in presenteeism research is based on analytical approaches such as linear regression or structural equation modeling to examine the relationship between presenteeism and its correlates. While these statistical models have been appropriate for the research questions addressed, they usually assume that presentees are a homogeneous group, which we are refuting. Therefore, if presentees are not a homogeneous group, how can count measures of presenteeism days be best used for understanding presentees’ experiences? The starting point is that there are heterogeneous groups of presentees since their performance and health are unlikely to be all affected in the same way by their work environment. In line with this, Ferreira et al. (2021) showed that blood markers (glycemia and CRP) affect productivity during presenteeism, thus supporting the idea that there are resources moderating the effect of presenteeism on performance or productivity. The association between presenteeism and its consequences (positive or negative) on performance and health (physical and mental) is still an area that is still largely unexplored and poorly understood. In two Taiwanese studies, no long-term effect of presenteeism on productivity and job performance (Lin et al., 2013; Zhou et al., 2016). Another study showed that presenteeism had a positive effect on innovative performance 6 months later when supervisor and colleagues support were high, but no effect on employee exhaustion (Chen et al., 2021). This points to the necessity to consider the subgroups of presentees beyond and above a count measure of the number of days of presenteeism. Indeed, two workers with the same health problem could report different number of days of presenteeism and show different levels of performance depending on the availability and relevance of different types of demands and resources. Despite being widely used, count measures of presenteeism alone may not capture variations in health and performance nor the conditions under which presenteeism could be functional. Yet, we can use count measures of presenteeism (and absenteeism) as a starting point for identifying broader groups of presentees.

### Different presentee profiles may experience different patterns of job characteristics

Presenteeism has been associated with a range of job characteristics, which in this case may act as stressors for presenteeism behavior, but it is unclear what job characteristics or stressors each type of presenteeism is linked to. According to Conservation of Resources theory (Hobfoll, 1989), behavior depends on workers’ resources as people strive to recover from resource loss, protect existing ones, or gain new resources. When facing stressors such as high job demands and poor working conditions, workers will capitalize on other resources available to avoid further resource loss or protect existing resources. Several studies have shown that presenteeism can be predicted by job insecurity (Heponiemi et al., 2010; Reuter et al., 2019), poor peer support (Gosselin et al., 2013) and managerial support (Mazzetti et al., 2019) work overload or job demands (Aronsson and Gustafsson, 2005; Biron et al., 2006; Miraglia and Johns, 2016), and work-family conflict (Johns, 2011; Arslaner and Boylu, 2017; McGregor et al., 2018). In their meta-analysis, Miraglia and Johns (2016) showed that job demands such as a high workload, negative relational experiences at work, and experienced stress at work were linked to higher presenteeism behavior. Some job characteristics or stressors have been found to have positive and negative associations with presenteeism. Aspects of job control such as decisional latitude, adjustment latitude, and skill discretion with presenteeism vary across studies and can possibly explain the weak correlation found by Miraglia and Johns (2016). Even social support is also sometimes positively associated with presenteeism, as workers do not want to let their colleagues down (Biron et al., 2006) or they decide to attend work as they find it therapeutic to be in a supportive family-like climate (Knani et al., 2021). Overall, job stressors have consistently been found to be related to mental (Duchaine et al., 2020) and physical health impairments (Gilbert-Ouimet et al., 2014) and increased presenteeism (Miraglia and Johns, 2016).

Therefore, to better understand the different presenteeism profiles, it is important to also understand how different job characteristics or stressors relate to different groups of presentees. For completeness, in addition to job characteristics we also explore whether the groups differ in sociodemographic characteristics (gender, age, and type of occupation), which is in line with previous studies showing certain work groups such as women and managers have higher presenteeism prevalence (Aronsson et al., 2000; Aronsson and Gustafsson, 2005). Our final proposition is therefore as follows.

*Proposition 3*: Different subgroups of presentees, identified based on their common health status and performance level, show different patterns in their exposure to job stressors.

### Study aims

Following the three propositions developed on the basis of the literature, the first aim of this study is to test the HPFP typology (Karanika-Murray and Biron, 2020) by substantiating the existence of quantitatively distinct profiles of employees who are ill but present at work, based on their reported common health problems and levels of performance. The second aim of the study is to investigate patterns of presenteeism and absenteeism among these profiles. The third aim is to evaluate differences among presentee profiles in terms of job characteristics or stressors that are typically associated with work-related health problems, whilst also characterizing these groups in terms of their demographic characteristics. Despite the interest in the HPFP model in the literature, no one to date has attempted to test it empirically. Before going further in the development of specific interventions for each profile as suggested by Karanika-Murray et al. (2021), it is important to test whether the model holds up. This proof-of-concept provides an empirical demonstration of how the model can be tested and raises questions about how future research can continue to advance it.

## Materials and methods

### Participants and procedure

A total of 205 employees from a large company in the UK private sector were invited to complete an online questionnaire on occupational stress and well-being. All worked in one business unit that was divided into two operational departments. From those, 159 gave their informed consent and completed the questionnaire, indicating a response rate of 77.6%. Among these 159 participants, a total of 108 (67.9%) reported at least 1 day of presenteeism over the last 3 months. We excluded two participants from the analyses due to incoherent response pattern (multivariate outliers). The final sample consisted of 106 participants who worked while ill at least 1 day during the past 3 months.

A broad range of job roles was represented including managers and senior officials (23.1%), professionals and technicians (5.6%), administrative and clerical staff (41.7%), sales and customer service staff (19.4%) and 10.2% of workers in basic occupations that require a minimum level of school education. The sample included 58.3% women. A total of 27% had a least one child under 18, and 48.2% were single whereas 49% had a partner (2.8% were divorced or separated). The majority of participants (92.6%) were in full-time employment. In terms of age distribution, 12% were under 21 years old, 59.3% were aged 21–30, 13.9% were 31–40, 7.4% were 41–50, and 6.5% were 51–60 years old, with just 1% of the sample aged above 60.

### Measures

#### Common health problems

Consistent with the HFPF (Kendall et al., 2016; Karanika-Murray and Biron, 2020), common health problems were assessed as *mental health*, which was measured with 11 items (e.g., constant irritability, tiredness, anxiety, difficulty concentrating, Cronbach’s α = 0.91), and *psychosomatic symptoms*, which were measured with 8 items (e.g., lack of appetite, insomnia, indigestion, α = 0.79) from the ASSET questionnaire (Cartwright and Cooper, 2002). Both scales considered the frequency of symptoms occurring over the past 3 months and were scored from 1 (never) to 4 (often). Normative data from the UK private sector was used to compare the sample in this study to the norms and to derive percentiles.

#### Performance

Consistent with the person-centered approach that allows using several combination of variables to evaluate the existence of subgroups of participants (Meyer and Morin, 2016), we measured three indicators of performance in combination. First, one item from the World Health Organization *Work Performance Questionnaire* (HPQ, Kessler et al., 2003) was used to measure subjective ratings of overall job performance over the past 28 days on a scale of 1 (worst) to 10 (top performance) (Kessler et al., 2003). Second, six items from the employee version of the HPQ were used to evaluate *quality of performance relative to other workers* (“How often was your performance lower than most workers on your job?”) and *quality of performance* (“How often was the quality of your work lower than it should have been?”) over the past 28 days (Cronbach’s α = 0.73). Third, *productivity* over the last 3 months was measured using an item from ASSET (Cartwright and Cooper, 2002): “Over the last 3 months, how productive have you felt in your job?” Participants responded on a 5-point percentage scale (1 = Less than 70% of the time; 5 = 100% productive). Second, in this study, although the term performance is used to concord with the terms used in the HPFP (Karanika-Murray and Biron, 2020), it includes both the within- (productivity) and between-person (relative performance) constructs. Note that all items of the performance indicators exclude health-related limitations. This is to differentiate health status from performance levels and is in line with our argument that presenteeism does not systematically and uniformly affect performance and productivity.

#### Absenteeism and presenteeism

Absenteeism was the number of days of absence from work during the last 3 months (i.e., “Over the last 3 months, how many working days have you been off work through illness or injury?”). Similarly, presenteeism was measured as the number of days the respondent came to work despite illness (i.e., “How many working days have you been coming to work even though you were ill or injured?”). Although many studies have used a 12-month period (Navarro et al., 2019), we reduced this to a 3-month interval to reduce recall bias. Several other studies have used a shorter recall period for the same reason (Knani et al., 2018; Ruhle et al., 2019). As suggested by Johns (2010), presenteeism and absenteeism were measured using an open ended fill-in-the-blank response format where respondents indicate the number of days they were absent or present, without suggesting categories to measure both absenteeism. This avoids a priming effect where categories of responses with specific range of days are presented to participants.

*Job characteristics* were measured using 37 items from ASSET (Cartwright and Cooper, 2002) including *work-life conflict* (e.g., “My work interferes with my home and personal life,” Cronbach’s α = 0.71), *low job control* (e.g., “I have little control over many aspects of my job,” α = 0.82), *poor work relationships* (e.g., “My relationships with colleagues are poor,” α = 0.85), *job insecurity* (e.g., “My job skills may become redundant in the near future,” α = 0.68) *unfair pay and benefits* (i.e., “Not as good as other people doing similar work,” one item), and *work overload* (e.g., “I set unrealistic deadlines,” α = 0.80). These items were scored from 1 (strongly disagree) to 6 (strongly agree).

### Analytical approach

To investigate the existence of subgroups, this study uses a person-centered approach. In contrast to the variable-based approach traditionally used in presenteeism research, instead of looking at relationships between variables the person-centered approach aims to “identify subpopulations presenting differentiated configurations (profiles) with regard to a system of variables” (p. 584). An advantage of the person-centered approach is that can focus on a system of variables, used in combination instead of considering them in isolation (Meyer and Morin, 2016). In this study, this system of variables includes two performance indicators and productivity, and common health problems by including both the psychosomatic and mental health scales.

We used latent profile analysis (LPA) to identify distinct profiles of presenteeism among respondents depending on their common health problems health and self-rated work performance. LPA is a model-based iterative method that defines classes of participants based on their common characteristics. The number of profiles are determined using an sequential process where classes are added until various indices (Akaike Information Criteria—AIC, Bayesian Information Criteria—BIC, entropy, class proportion < 5%, Bootstrapped Likelihood Ratio Test—BLRT) indicated the best fit to observed data (Nylund et al., 2007). Since there are no objective cut-off scores for the fit statistics, the best model was selected according to the following criteria: lowest BIC (suggesting best parsimony), highest entropy (suggesting distinct non-overlapping profiles), and non-significant BLRT test (suggesting that no additional profile is needed to improve fit). Additionally, the conceptual meaning of the iteratively derived solutions was used to select the best profile structure (Foti et al., 2011).

Latent profile analysis was conducted using Mplus 7.0 (Muthen and Muthen, 2017) with continuous (normal) indicators. To complement the main analyses, one-way ANOVAs with REGW *post-hoc* comparisons and chi-squared, were used to compare the latent profiles on a series of indicators using SAS 9.4 (SAS Institute, 2014) and conventional alpha level of 5%.

**FIGURE 2:**
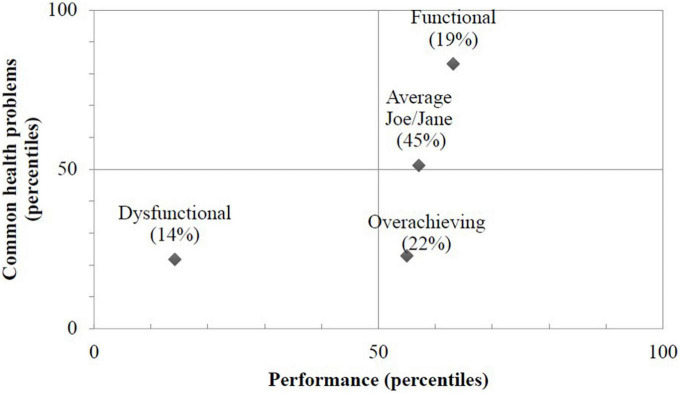
Four latent profiles of presenteeism according to performance level and common health problems.

To create a visual illustration of the presenteeism typology (Figure 2), indicators of common health problems and performance dimensions were (1) converted into percentiles, (2) averaged within each dimension, and (3) displayed in a X-Y dot plot. Performance percentiles were computed according to sample means while, for mental health, percentiles were computed according to normative data (UK private sector).

## Results

### Latent profile analyses

Solutions for latent profile models ranging from 1 to 5 profiles were investigated in the 106 participants (see Table 1). The lowest BIC (2233.97) supported a 3-profile solution while entropy was maximal for the 5-profile solution (0.86). Bootstrapped likelihood ratio test (BLRT) was still significant for the 4-profile solution, χ^2^(*df* = 6) = 22.54, *p* = 0.000, suggesting that additional profiles may be added. However, trivial profile comprising only 1 participant was observed for 5-profile solution and the BLRT test was no longer significant (*p* = 0.06). Hence, based on these observations and its interpretability, the 4-profile solution was retained.

**TABLE 1:** Fit indices for latent profiles analyses (*N* = 106).

Number of profiles	Parameters	LL	AIC	BIC	Entropy	BLRT
1	10	−1134.17	2288.34	2314.97	1	–
2	16	−1091.08	2214.17	2256.78	0.81	86.17***
3	22	−1065.69	2175.38	2233.97	0.82	50.79***
4	28	−1054.42	2164.83	2239.41	0.81	22.54***
5	34	−1044.84	2157.68	2248.24	0.86	19.15 (*p* = 0.06)

LL, log-likelihood; AIC, Akaike information criteria; BIC, Bayesian information criteria; BLRT, Bootstrap likelihood ratio test. ***p < 0.001.

Estimated standardized (%) means on each of the health and performance indicators according to the 4 profiles are displayed in Figure 2. The first profile comprised 19% of the sample (*n* = 20) and was termed *Functional presenteeism*. It refers to workers who report a higher-than-average performance and a better mental health (low scores on mental health and psychosomatic problems scales). The second profile comprised 14% of the sample (*n* = 15) and was termed *Dysfunctional presenteeism* because it comprised individuals with lower-than-average health and performance indicators. The third profile, comprising 45% of the sample (*n* = 48), was labeled *Average Joe/Jane* and represented sickness presentees with and average health with average performance indicators. The last profile included 22% of the sample (*n* = 23) and comprised individuals with a substantially poorer health, but who manage to maintain somehow a relatively good (average) performance. Participants in this category were referred to as *Overachieving presentees* given that manage to maintain their performance level relatively high, but they do so at the expense of their own health.

The four presentee profiles based on the two dimensions (performance and health) are displayed in Figure 2. Functional, Overachieving, and Dysfunctional are in their expected positions in each quadrant, but Therapeutic was not where expected. This profile is supposedly characterized by poor performance and relatively good health, but no one corresponded to this combination. Instead, a group representing an average performance and average health was found.

### Attendance patterns across presenteeism profiles

Profiles were first compared in terms of the average number of days of reported presenteeism and absenteeism over the last 3 months using a generalized linear model for over-dispersed count data (negative-binomial distribution). Results suggest that Average Joe/Jane presentees reported a significantly lower number of days of presenteeism over the past 3 months (8.10 days) compared to presentees in the other three profiles (Functional = 15.20 days, Dysfunctional = 16.40 days, and Overachieving = 14.39 days), *F*_(3,102)_ = 2.60, *p* < 0.05. There was no significant difference in the number of days of absenteeism over the past 3 months across the four profiles (Functional = 2.20 days, Dysfunctional = 2.73 days, Average Joe/Jane = 3.30, and Overachieving = 2.48 days), *F*_(3,102)_ = 0.40, *p* = 0.75. Table 2 displays the average number of days of presenteeism and absenteeism for each of the profiles.

**TABLE 2:** Estimated means (standard errors) for the common health problems and performance indicators for each of the four presenteeism profiles (*N* = 106).

	Presenteeism profiles			
	
Overall prevalence (n)	Functional (*n* = 20)	Dysfunctional (*n* = 15)	Average Joe/Jane (*n* = 48)	Overachieving (*n* = 23)
Productivity	4.3 (0.2)	1.5 (0.3)	3.2 (0.1)	3.0 (0.2)
Performance (quality)	4.1 (0.1)	3.2 (0.1)	4.2 (0.1)	4.3 (0.1)
Performance (overall)	8.1 (0.3)	5.5 (0.4)	8.3 (0.2)	8.2 (0.3)
Psychosomatic symptoms	13.9 (0.7)	32.1 (0.9)	20.8 (0.5)	31.1 (0.7)
Mental health	9.6 (0.6)	18.3 (0.7)	15.4 (0.4)	18.6 (0.6)

These comparisons were also performed using categories of presenteeism (1–7 vs. 8–30 vs. 30 days or over in the last 3 months) and absenteeism (0 vs. 1+ days in the last 3 months). Results showed a significant difference in the frequency of presenteeism across profiles, χ^2^(*df* = 6) = 13.44, *p* = 0.04. A higher proportion of Functional (85%) and Average Joe/Jane (81%) reported working ill between 1 and 7 days over the past 3 months, whereas these proportions are lower in the Dysfunctional (53%) and Overachieving (65%) profiles. No significant difference was found between profiles for absenteeism categories, χ^2^(*df* = 3) = 6.05, *p* = 0.11.

### Job stressors and individual characteristics across presenteeism profiles

**FIGURE 3:**
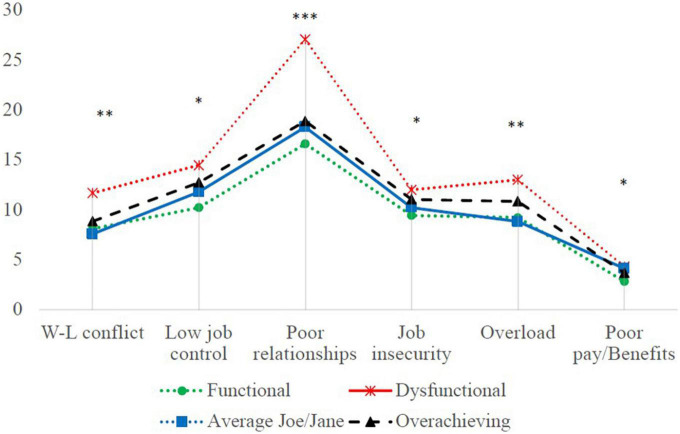
Differences in exposure to job stressors according to four latent profiles of presenteeism. *p < 0.05, ^**^p < 0.01, ^***^p < 0.0001.

One-way ANOVAs were performed to compare job characteristics across presenteeism profiles (see Table 3 and Figure 3). Results revealed significant differences for most variables. Specifically, the Dysfunctional profile reported significantly higher exposure to stressors related to work-life conflict (*M* = 11.47 vs. 7.77–8.74), poor work relationships (*M* = 25.73 vs. 16.60–20.09), job insecurity (*M* = 12.33 vs. 9.15), and work overload (*M* = 12.47 vs. 8.90–9.08) (all *p*’s < 0.05). Dysfunctional and Overachieving presentees both showed higher exposure to low job control compared to the other two profiles (*M* = 13.53 and 13.57 vs. 10.00). Finally, for unfair pay and benefits, Dysfunctional and Average Joe/Jane (*M* = 4.20 and 4.15 vs. 2.75) profiles exhibit higher scores compared to the Functional profile.

**TABLE 3:** Analysis of attendance, job stressors, attitudinal, and socio-demographic characteristics for the four profiles.

Means (standard error)	Functional (*n* = 20)	Dysfunctional (*n* = 15)	Average Joe/Jane (*n* = 48)	Overachieving (*n* = 23)	*F*_(3,102)_
**Attendance**					
Presenteeism (#days)	15.20 (3.89)_a_	16.40 (4.84)_a_	8.10 (1.37)_b_	14.39 (3.44)_a_	2.60*
**Presenteeism**					
1–7 days	85% (17)	53% (8)	81% (39)	65% (15)	13.44*
8–30 days	0% (0)	27% (4)	15% (7)	22% (5)	
>30 days	15% (3)	20% (3)	4% (2)	13% (3)	
Absenteeism (#days)	2.20 (0.76)	2.73 (1.08)	3.30 (0.72)	2.48 (0.79)	0.40
**Absenteeism**					
0 day	50% (10)	40% (6)	23% (11)	44% (10)	6.05
1+ days	50% (10)	60% (9)	77% (37)	56% (13)	
Ratio hours worked to hours contracted	1.02 (0.09)_b_	1.34 (0.10)_a_	1.03 (0.06)_b_	1.04 (0.08)_b_	2.65*
**Job stressors**					
Work-life conflict	8.05 (0.78)_b_	11.47 (0.90)_a_	7.77 (0.50)_b_	8.74 (0.73)_b_	4.46**
Low job control	10.00 (0.99)_b_	13.53 (1.14)_a_	11.90 (0.64)_ab_	13.57 (0.92)_a_	2.90*
Poor work relationships (colleagues and superior)	16.60 (1.43)_b_	25.73 (1.65)_a_	18.23 (0.92)_b_	20.09 (1.34)_b_	6.79***
Job insecurity	9.15 (0.65)_b_	12.33 (0.75)_a_	10.35 (0.42)_ab_	10.96 (0.61)_ab_	3.63*
Unfair pay and benefits	2.75 (0.39)_b_	4.20 (0.45)_a_	4.15 (0.25)_a_	3.87 (0.36)_ab_	3.30*
Work overload	8.90 (0.80)_b_	12.47 (0.92)_a_	9.08 (0.52)_b_	11.17 (0.75)_ab_	4.87**
**Socio-demographics^**1**^ (%)**					X^2^ (df = 3)
Managers/Professional	20.0_a_	46.67_b_	14.58_a_	34.78_ab_	8.06*
Gender (% female)	45.00	46.67	62.50	69.57	3.84
Age (% >30)	20.00	20.00	27.08	43.48	3.83

^1^Categorical variables: % in each cell are indicated. Subscripts letters indicate differences among profiles. All perceived job stressors are scored so that higher means imply a higher exposure to each stressor. *p < 0.05, **p < 0.01, ***p < 0.001.

For demographics, there was a significantly higher proportion of managers and professionals in the Dysfunctional profile (46.67%) compared to Functional (20%) and Average Joe/Jane (14.58%). Gender and age group did not differ across profiles.

## Discussion

This proof-of-concept study aimed to validate the quantitatively distinct profiles of presenteeism as a function of self-rated performance and common health problems. Proposition 1 was supported given that four distinct profiles of presenteeism were identified in this group of presentees, which is in line with the proposition by Karanika-Murray and Biron (2020) and the HPFP. Functional presentees reported fewer common health problems and higher performance than Dysfunctional presentees. Although Overachieving and Dysfunctional presentees reported comparable levels of common health problems, Overachieving presentees had higher self-rated performance levels than Dysfunctional presentees. Despite differences in the severity of their health ailments, the performance level of Overachieving, Average Joe/Jane and Functional presentees were similar. The fourth profile was labeled as Average Joe/Jane presenteeism, but as a separate category it represented nearly half of the sample (45%) with average health and performance levels. Although labeled as average, they are very similar to Functional presentees in terms of exposure to job stressors, with the exception that they reported experiencing more unfair pay and benefits and poorer health. This was reflected by their relative position in the quadrants (Figure 2) and their scores on psychosomatic symptoms and mental health (Table 2) which are lower compared to Functional presentees. The presence of the Average Joe/Jane profile with a rather large percentage of participants in it is possibly an artifact of the statistical technique. However, it is also reasonable to think that it reflects reality: It is unlikely that workers are distributed in four completely distinct and watertight quadrants, which would be tantamount to saying workers are only at the extremes of the health and productivity continuum and not at the center. It is more likely to think that for many, working with a minor health problem is a rather common occurrence and future research should investigate in what context and with what resources can help workers strike the right balance between performance demands and their health constraints. Alternatively, we could have defined the four quadrants *a priori* and classified participants in one of them based on their health-performance scores. This would imply that that the Average Joe/Jane profile would be left empty since it would not exist. The problem with this approach is that the demonstration of the existence of distinct presenteeism profiles would be created by the researchers and not driven by the data.

According to the original HPFP conceptualization, there should have been a Therapeutic profile which would include presentees who find refuge in work and who, despite a relatively good health, also show poor performance. Although we did not find any participants corresponding to Therapeutic presenteeism in this specific sample, future studies with a larger sample and also varying types of job roles should further investigate this type of presenteeism and the four configurations.

In line with Proposition 2 on differences in attendance behavior, we found that the average number of days of presenteeism was similar across Functional, Dysfunctional, and Overachieving profiles (14–16 days) whereas Average Joe/Jane presentees reported half (8 days) that number of days of presenteeism. This highlights a current problem in research when presenteeism is measured as a count, as in the number of days or times the person works through illness, without considering the severity of the health ailment or the way their performance is affected. Indeed, although Functional and Dysfunctional profiles are highly contrasted both in terms of common health problems and performance levels, they report a comparable number of days of presenteeism. Demonstrating the existence of differences in attendance patterns among profiles of presentees is in line with Gerich’s (2015) recommendation to disassociate the effects of the health component from the decision component in presenteeism research. When looking at presenteeism days as categories, higher proportions of Functional (85%) and Average Joe/Jane (81%) presentees came to work ill between 1 and 7 days, compared to Dysfunctional (53%) and Overachieving (65%). This is line with previous studies showing that presenteeism is closely related to the severity of the health ailment (Caverley et al., 2007). Surprisingly, however, we found no difference in absenteeism among the profiles, which is counterintuitive since poorer health issues would imply the need to take sick leave. This is certainly something to explore further, with a more detailed examination of health conditions and performance requirements and/or types of jobs and work environments.

As for proposition 3, several differences were found among profiles in terms of perceived job stressors. Dysfunctional presentees report systematically higher exposure to all stressors compared to other profiles, in particular Functional presentees. Surprisingly, few differences were found between Functional, Average Joe/Jane, and Overachievers (those three profiles that define the health dimension with relatively good performance), suggesting that job stressors did not discriminate among these three profiles despite the presentees’ differences in terms of common health problems. Tentatively, this could be explained by the fact that the sample comprised only workers who declare themselves as presentees by reporting at least 1 day of presenteeism over the past 3 months. In the general population, these job stressors have been consistently shown to be predictive of common health problems (Duchaine et al., 2020). It is also likely that there are other moderators affecting the consequences of presenteeism. Lu and Cooper (2022) recently highlighted that there are moderators intervening in the presenteeism-outcome relationship. Their longitudinal study showed that over a 5-month period, long-working hours increased presenteeism, which in turn had a negative effect on performance but only for employees with low and intermediate intrinsic work value orientation, or in other words, those who value their job for its intrinsic factors such as feeling autonomous or competent, instead of for its extrinsic factors such as financial and social rewards. The association between presenteeism and performance was not significant for those with high intrinsic work orientation. As highlighted by Karanika-Murray and Biron (2020), presenteeism is a dynamic process that involves an interaction between individuals, their working environment, and the broader context, and that its consequences (positive and negative) can co-occur. Our results also reflect the study by Bergström et al. (2020) who showed that despite having negative effects on health in the long-term, working while ill in a resourceful environment can buffer its consequences.

Overall, this study highlights how subgroups of presentees, despite similar attendance patterns, can have very different exposure to stressors and access to resources to protect their health and their performance. This calls for broadening the scope of presenteeism to include more person-centered as well as more process-oriented studies to understand how presenteeism behavior unfolds overtime. The adaptive function of presenteeism is a choice that is made under tension for allocating resources/avoiding loss of resource at work under health constraints. This tension is exacerbated by a stressful work environment, which tends to deplete both health and performance resources. The resourceful work environment was more closely associated with the Functional presentee profile.

Finally, in terms of sociodemographic characteristics, the results showed that managers and professionals were more likely to be in the dysfunctional profile, namely maintaining a high level of performance at the expense of their health compared to other job categories. Previous studies have also found a higher prevalence of presenteeism and presenteeism propensity in managers and professionals compared to workers in other occupations (Kinman, 2019; Reuter et al., 2019). This is in line with the suggestion by Ruhle et al. (2019) to conduct more research on presenteeism in specific sectors and job types, given that there have been so far very few comparative studies. Our results suggest it would be particularly relevant to investigate presenteeism profiles across various occupations and sectors.

### Contributions

At the theoretical level, this proof-of-concept study concurs with the HPFP (Karanika-Murray and Biron, 2020) to suggest that there can be a bright side to presenteeism, that of Functional presenteeism, and that heterogenous groups exist within presentees. The view of presenteeism as a strictly negative phenomenon obscures its positive adaptive potential for individuals. This bright sight appears to depend on the work context and the individual’s resources to accommodate health and performance requirements in tandem. But it is important to extend this proof-of-concept study with other and larger groups in the working population. Importantly, this study can help us to move toward addressing the scarcity of research investigating interventions to better manage presenteeism in such a way as to preserve individuals’ health and protect their performance. To better manage presenteeism, interventions ought to be tailored to the workers’ needs. Our study suggests these needs might differ across profiles and that specific resources must be made available and used to manage presenteeism more efficiently.

These resources can vary but should be tailored according to the profile. Mori et al. (2022) conducted a study with 15,158 non-managerial workers from 7 companies that are actively engaged in health promotion activities in Japan. They used the quality and quantity (QQ) method to calculate a presenteeism score based on the extent to which a health impairment is present or not and if affected their work. Based on health impairments that were perceived as affecting their work, participants then describe the quantity and quality of their work when they were experiencing the health problem compared with when they had no problems on a scale of 0 (unable to work) to 10 (normal). The presenteeism score is then computed by subtracting the quantity and quality impacts (ranging from 0 to 10) from 100. The superior quintile is defined as presenteeism. Their results show that (1) there is a relationship between presenteeism and perceived supervisor support for health (2) that even after adjusting for psychological distress and work engagement, this relationship is weakened but still significant. This suggest that beyond health impairments and performance demands, different types of resources come into play and influence presenteeism. It is important to note that their support for health item is in fact managerial support for both health and performance (i.e., “My supervisor supports employees to work vigorously and live a healthy life.”) Although vigor at work is measured in engagement scales (Schaufeli et al., 2006), it is also embedded in performance measures. In their study (Mori et al., 2022), higher presenteeism was associated with lower supervisor support for health. This suggest that managerial support is perceived as a resource which reduces presenteeism *via* psychological states.

However, as Mori et al. (2022) rightly point out, there are other factors influencing presenteeism. Karanika-Murray and Biron (2020) suggest several types of individuals, group, managerial, and organizational resources that can affect presenteeism, and PSSH is one of them. Through encouraging employees to take care of their health, providing flexibility in managing work hours and the content/quantity of work, managers have an influence on health, and ultimately on productivity. By affecting these two dimensions, they can elicit different presenteeism profiles in their employees. In the same vein Ammendolia et al. (2016) conducted a study in a large Canadian finance company using a step mapping approach to design multi-pronged intervention program to reduce presenteeism. Since mental health was the most prominent health issue in the organization, it was the focus of their action plan. However, as the authors state, they found limited evidence from the scientific literature on effective interventions for reducing presenteeism. Their interventions were therefore based on the experiences and opinions of the participants. In this paper, we suggest that such programs could be tailored to meet the specific needs of presenteeism profiles. Interventions for dysfunctional presentees would have to prioritize the more severe health issues, whereas interventions for functional presentees would focus on resources to preserve health and performance or improve them so that presenteeism is no longer required (optimal health) (Karanika-Murray et al., 2021).

At the methodological level, this study highlights the appropriateness of a person-based approach, as it suggests that not all profiles are exposed to the same constraints. In addition, it concurs with previous researchers criticizing the conceptualization of presenteeism as productivity loss (e.g., Ruhle et al., 2019). The way productivity and performance are affected during illness is likely to vary depending on the health ailment, the work situation, or occupation, for example. Take for example a knowledge worker who is suffering from depression but is also in denial of that, who would work every day for 3 months. It is likely that this worker would not report a high number of days of working with illness but would probably be less productive than usual. However, if instead of depression, this knowledge worker suffered from a fractured leg, productivity loss would probably be lower, but the number of days of presenteeism would be high given the timeframe required to heal a fracture. It is likely that the relationship between health and performance is an idiosyncratic evaluation that only the presentee him/herself can make to decide if it is better to work or take leave, but colleagues, managers, and organizations can support more specifically and provide resources to support health and performance in tandem. This idiosyncratic evaluation is worth exploring further, as Lohaus and Habermann (2021) and Whysall et al. (2023) have sketched, by examining the decision-making process and trade-off considerations that presentees make.

The study also brings together two dimensions currently used to investigate presenteeism, namely health and performance, and disentangles them from the total amount of presenteeism days. Several studies have found that sickness presenteeism and absenteeism are correlated (Miraglia and Johns, 2016) and it has been argued that this reflects the severity of the health ailment. Considering that one of the problems of measuring presenteeism as a count measure (number of days or time) is that it simultaneously captures the tendency to choose presenteeism over absenteeism while ill, or presenteeism propensity (Gerich, 2016) along with the number of health problems, namely the person’s vulnerability (Ruhle et al., 2019). Indeed, an individual declaring several days of presenteeism over a certain period is likely to be in a poorer health compared to an individual with a lower number of days. The HPFP allows to separate the health and performance factors from the count measure of presenteeism, thus disentangling the three phenomena.

### Limitations

As this was a preliminary proof-of-concept study, there were some limitations. First, although adequate for the type of analyses, the sample was small and specific to one company in one country, and it is possible that a larger sample from the broader workforce will allow to detect different configurations. Perhaps a larger sample would reveal a profile that would be closer to what the HPFP defined as Therapeutic presenteeism, and for which there is evidence in qualitative studies. This is the purpose of a proof-of-concept study, namely, to explore and test ideas in order to evaluate if the original proposition by Karanika-Murray and Biron (2020) stands with a small sample before investing more substantial resources in a major project. Another limitation is the study’s cross-sectional design. The presenteeism literature is still weak on research using longitudinal designs with several time-points. A larger study is currently underway with a population-based sample and 4 waves of measurements collected during the COVID-19 pandemic. This will allow, among other things, to understand what fosters functional presenteeism, how the behavior unfolds as health and performance configurations change, and how to prevent dysfunctional presentees from further deterioration or from becoming absent.

## Conclusion

Identifying the existence of subgroups of presentees and exploring differences between them can open important new avenues for research and interventions to promote both better health and performance concurrently through. As suggested by Karanika-Murray et al. (2021), once the decision to work ill is made, there must be an assessment of the worker’s needs in terms of available resources and task adjustments. This assessment often involves the manager, who must be properly trained to support the worker in order to facilitate a return to more functional presenteeism, or even a return to more optimal health and performance. Organizational policies also need to be clear about what is legitimate and expected from workers when they experience health problems (Ruhle and Süß, 2020). Understanding the conditions in which presenteeism could be a functional and sustainable choice would be particularly useful, considering that work is good for health and well-being (Waddell and Burton, 2006). As Meyer and Morin (2016) highlight, person-centered approaches are complementary to traditional variable-based ones but have hardly been used in the field of presenteeism. Similar to studies of absenteeism showing different trajectories of sickness absence (Hallman et al., 2019), future research could consider trajectories of presenteeism and identify the mechanisms behind them.

## Data availability statement

The raw data supporting the conclusions of this article will be made available by the authors, without undue reservation.

## Ethics statement

The studies involving human participants were reviewed and approved by the Ethics Committee of Lancaster University. The patients/participants provided their written informed consent to participate in this study.

## Author contributions

HI provided statistical support and conceptual guidance. All authors participated in the revision process and approved the submitted version.
